# Toward A Greater Understanding of the Ways Family-Based Treatment Addresses the Full Range of Psychopathology of Adolescent Anorexia Nervosa

**DOI:** 10.3389/fpsyt.2019.00968

**Published:** 2020-01-24

**Authors:** James Lock, Dasha Nicholls

**Affiliations:** ^1^Department of Psychiatry and Behavioral Sciences, Stanford University School of Medicine, Stanford, CA, United States; ^2^Division of Psychiatry, Department of Brain Sciences, Imperial College London, London, United Kingdom

**Keywords:** family-based treatment, anxiety, cognitions, social, family

## Abstract

Family-based treatment (FBT) for anorexia nervosa (AN) is an empirically supported treatment for this disorder. Derived from several different schools of family therapy, it is a highly focused approach that initially targets weight restoration under parental management at home. However, the view that manualized FBT is solely a behavioral therapy directing parents to refeed their children AN with the single purpose of weight gain is a common but misleading over simplification of the therapy. Indeed, weight restoration is the main goal only in phase 1 of this 3-phase treatment. When practiced with fidelity and skill, FBT's broadest aim is to promote adolescent development without AN thoughts and behaviors interfering and disrupting these normal processes. Although weight restoration is a key starting point in FBT, the entire course of treatment takes into consideration the ongoing impact of starvation, cognitions, emotions, and behaviors on adolescent development. These factors associated with maintaining low weight are viewed in FBT as interfering with the adolescent being able to take up the tasks of adolescence and thus must be overcome before fully turning to those broader adolescent tasks. In addition, FBT recognizes that adolescence takes place in the context of family and community and respects the importance of learning in a home environment both for weight gain as well as related developmental tasks to have a lasting effect. Specifically, in this article we describe how the current FBT manualized approach addresses temperament/personality traits, emotional processing, cognitive content and process, social communication and connections, psychiatric comorbidity, and family factors. This report makes no claim to superiority of FBT compared to other therapies in addressing these broader concerns nor does it add interventions to augment the current manual to improve FBT.

## Introduction

The view that manualized family-based treatment (FBT) is solely a behavioral therapy directing parents to refeed their children with anorexia nervosa (AN) with the single goal of weight gain is a common but misleading over simplification of the therapy (1). Reports suggest that therapists implementing FBT are concerned with the lack of specific interventions in the approach to address issues such as psychiatric comorbidity, perceived family dysfunction, and broader adolescent development and functioning (2–5). While there is deliberate focus on weight and eating normalization, FBT aims more broadly at promoting adolescent development without AN thoughts and behaviors interfering and disrupting these processes. Thus, in the structure, intervention, therapeutic style, and treatment phases, FBT attends to the broad psychopathology of AN. However, in FBT, weight restoration is a key starting point and is approached from a developmental and learning perspective that recognizes the realities of adolescent abilities and the importance of their home environment for weight gain to have a lasting effect. In this article we review how FBT promotes weight restoration and in this context, temperament/personality traits, emotional processing, cognitive content and process, social communication and connections, psychiatric comorbidity, and family factors.

There are only limited data that support the view that manualized FBT leads to improvements in psychosocial functioning and related psychopathology. For example, longer term outcome studies found high levels of psychosocial functioning 4 years posttreatment with FBT (6). With a mean age of about 19 years at time of follow-up, 73% of the participants were no longer in any psychiatric treatment and 73% were in full time school or work. In addition the Youth Self Report scale (7) scores improved from 40.6 at baseline (BL) to 25.6 at 1 year posttreatment and the Child Behavior Checklist (7) scores improved by over 20%. In a separate study Agras (8) and colleagues found (unpublished results) that for those treated with FBT the Beck Depression Inventory (BDI) (9) improved from 14.59 a BL to 7.1 at 1 year follow-up. Furthermore, the Rosenberg Self-Esteem Scale (10) improved from.4.2 at BL to 2.1 at follow-up. In addition, the Quality of Life and Satisfaction and Enjoyment Questionnaire (Short) (11) improved from 47.8 at BL to 53.2 at follow-up. There were also reductions in the Self-Esteem Questionnaire—Anxiety subscale score (4.5 at BL to 4.1 at follow-up) and the Childrens' Yale-Brown Obsessive Compulsive Scale (12) (3.82 at BL to 3.64 at follow-up). Interpretation of these findings is limited by the fact that these secondary outcomes were not tested for statistical significance nor were the studies powered to examine them. In addition, there was no evidence that these changes were greater in FBT than comparison treatments.[e.g., Adolescent Focused Therapy (13) and Systemic Family Therapy (14)]. A recently published qualitative study also support the view the FBT is helpful in overall adolescent development (15). Thus, these data are provided only for descriptive purposes to illustrate that FBT appears to have positive effects on broader AN psychopathology.

The treatment stance, family context, agnostic view of AN, prioritization of AN over other psychiatric issues, as well as the emphasis on adolescent development provide the overall context for how FBT helps with the other associated problems that are often present. Much of the early emphasis in FBT is about empowering parents because AN is seen as life threatening while also limiting the adolescent's capacity for making sound decisions about eating, exercise, and weight gain (16). Thus, parents are needed to temporarily manage these issues, but only until the adolescent herself is again able to make reality based decisions about her health. Many anxieties and obsessions are directly attributable to weight loss and starvation and dissipate to a significant degree with weight gain in the home environment. Parents are not viewed as a necessary evil but rather the natural and best resource for helping their children with their problems as they navigate them during treatment. In this sense, the other problems that are common in AN such as social anxieties, mood lability, and family conflicts are seen as part of the family work in FBT. Initially, these problems are addressed mostly in the context of food and eating, but during phases 2 and 3 when the adolescent is more involved in her usual social, academic, and family life, support for taking up adolescent process around peers and family are a clear focus—that is indeed the reason for phases 2 and 3. While progress in phase 1 is highly predictive of long term outcome (17), this does not imply that phases 2 and 3 are not needed; instead, phase 1 is the foundation for the work of these phases.

It is also worth noting in this description of how FBT addresses associated problems common in adolescent AN is the important role of the individual one to one time that occurs at the beginning of every FBT session. During this approximately 10-min time when the therapist meets alone with the adolescent, there is an important and ongoing opportunity to build rapport, evaluate cognitive and emotional states, identify specific concerns the adolescent may be experiencing, and provide support. In phase 1 of FBT, these one to one meetings may be brief and consist mostly of taking the adolescent's weight and assessing her reactions to weight change and to the efforts her parents are undertaking to help her. Rapport is usually slow to build with adolescents with AN who, because of their investment in maintaining AN, initially perceive the therapist as obstructive. However, the skilled FBT therapist demonstrates consistent warmth, interest, and concern for the adolescent, particularly in those facets of her identity and behavior that are independent of AN. Studies suggest that therapeutic alliance is good in FBT, despite the often difficult beginnings (18, 19). This increased rapport built carefully over phase 1 typically improves in phases 2 and 3 as trust develops that issues will be respectfully addressed with the family. Material gathered in these brief sessions are essential for addressing cognitive, emotional, social, and family processes more broadly as FBT proceeds.

## Specific Strategies Employed in FBT to Address the Broad Behavioral and Psychopathology of Adolescent AN

### Weight Restoration

Many of the behaviors associated with AN can be attributed to the so called “starvation syndrome” characterized so well in the classic Minnesota Semi-starvation studies (20). It has been suggested that weight restoration should resolve much of the related psychopathology of AN and for many years inpatient settings saw this as a core treatment aim, only to find their patients relapsed on discharge.

One of the hallmarks of FBT is the early and highly focused encouragement of parental management of their child's eating while living with them in the home environment. Weight restoration in this context differs from weight restoration in a hospital or any other professionally managed environment. Home is the environment where normal eating and weight management is generalizable. FBT takes seriously the principle that for learning to be optimized, it must take place in the environment where the learning is relevant. There is good evidence that FBT's home learning environment is effective for helping adolescents with AN to eat and gain weight more quickly than other outpatient approaches, leading to both reduced hospital use and costs (8, 21, 22).

In addition to emphasizing generalization of behavioral learning, FBT also considers the developmental capacities of an adolescent, usually around the age of 14–15 years, to make sound decisions and manage eating (23–25). It is not expected nor typical for most adolescents at these ages to be in full control of what they eat—they do not earn money to buy food, generally do not regularly prepare food, nor do they eat special individualized meals; instead, parents have this responsibility in the context of family life. FBT's approach returns to the parents the authority for their adolescent's food intake, a responsibility that AN tries to take from them. FBT takes the position that this is not seen as a regression to an earlier developmental age but rather a result of AN, and uses externalization strategies to emphasize this point. During phases 2 and 3, as AN symptoms abate, adolescent processes either onset (for younger patients) or are reestablished. This is of all the more importance when the child is seriously malnourished and unable to participate meaningfully in making these decisions because of the obsessional anxieties associated with an irrational fear of weight gain (26). Nonetheless, it is important to note that it is during the first phase of FBT only that weight restoration is the almost exclusive overt focus. This phase lasts about 6–10 weeks and is followed by 2–6 months of FBT aimed at age appropriate management of eating during phases 2 and 3.

### Temperament and Personality Traits

A number of temperament or personality traits are associated with AN. The best characterized temperamental traits of relevance to AN are perfectionism (27–29), harm avoidance (30), (low) novelty seeking and persistence, traits that clinicians recognize can influence the course of illness, and treatment response. However, harm avoidance is significantly lower, and reward dependence significantly higher in individuals who recover from AN than in those who remain ill (31), suggesting that what appear to be traits may in fact be features of the illness. From a developmental perspective, an anxious temperament interferes with necessary adolescent social development with peers and associated socialization processes. FBT's approach to helping with this problem takes place in differing ways depending on the phase of treatment. In the weight restoration phase (phase 1), most anxieties are focused on food and eating, and in FBT support for managing these anxieties comes principally from parents as would be the case for most adolescent problems. In this phase, the adolescent gradually experiences decreased anxiety about weight gain by continuous exposure to food, eating, and weight gain (weekly weighing) (32). This prepares her for experimenting and testing her own anxieties about eating and weight gain during phase 2. During phase 2, socialized eating—at school, with friends, in restaurants, at parties—is a main focus. Parents are asked to help facilitate and support the adolescent in these exposures, with the aim of increased mastery of social anxieties in these situations a main treatment target. Helping the adolescent manage more general social anxieties is a treatment goal in phase 3 of FBT. In this phase, eating and weight restoration are not the focus, but instead, dating, sexuality, peer group participation, and individuation from parents are targeted. If at the conclusion of FBT there continues to be significant social anxiety, then additional treatment for these problems should be considered.

Perfectionism is common in AN and interferes with psychosocial functioning and may need intervention in its own right, as has been suggested by a modular addition to transdiagnostic CBT (33). In the context of adolescents with AN, an anxious temperament is, as noted above sometimes expressed as social isolation and avoidance. Perfectionism differs from social anxiety, although both may be partly based in an anxious temperament. Some of the ways perfectionism expresses itself in AN is through rigid control over eating, mealtime rituals, strict rule following, obsessional checking of calories, weight, fat grams, etc., and weighing (34). Data suggest that the perfectionism in this sense is highly related to obsessive compulsive personality features in AN (35).These, in turn, interfere with treatment response in FBT by making the task of refeeding and weight restoration in phase 1 even more challenging (36). Of interest, perhaps, is the fact that inpatient treatment may reinforce these perfectionistic and obsessive tendencies, reducing response (37). FBT in phase 1 approaches perfectionism behaviorally, that is through parental strategies that disrupt the ability to reinforce perfectionistic and obsessional behaviors related to food and weight (e.g., calorie counting, ritualized eating, excessive weighing, and mirror checking). These expressions of perfectionism as it relates specifically to AN are then the initial FBT target. In phase 2, perfectionism may contribute to social anxieties with peers by setting unrealistic standards for appearance and behavior either for the adolescent herself, for her peers, or for both (38). During phase 2, although weight gain is still an aim, there is an explicit focus on addressing adolescent experiments with eating and socializing with others. During the individual meeting between therapist and adolescent that begins every FBT session, the therapist has an opportunity to identify perfectionism and develop strategies with the adolescent to address these anxieties. These are then brought to the parents in the family session to address their impact and ways they can help overcome them—especially with comparison with peers around eating, food, weight, and exercise. During phase 3, perfectionism when present can be addressed in FBT as a problem that interferes with the adolescent's relationships and general self-esteem (39, 40). If perfectionism continues to be at a concerning level at the end of FBT, addition stand-alone treatment for this might be indicated outside the frame of FBT itself.

### Emotional Processing

Emotional difficulties are often implicated in the development or maintenance of eating disorders and may be a risk factor for their development (41). There are problems with experiencing emotions, emotional sensitivity, and emotion regulation. Alexithymia, the inability to identify and label emotions (42), is present to a significant degree across studies in patients with AN (43). Depression and anxiety are well-described antecedents as well as comorbidities of AN, with the suggestion that starvation blunts the experience of negative affect and is therefore inherently rewarding through the avoidance of aversive feelings (34, 44). Unlike emotional sensitivity, emotional regulation is conceptualized as a skill that is learnt throughout life and most especially during adolescence as many new emotions are first experienced (45). The process of effective emotion regulation is predicated on recognition of emotions, utilization of cognitive or other strategies to manage emotions, and the deployment of reflective function and emotional processing to enhance learning (46). Cognitive strategies commonly used for emotion regulation include acceptance of emotions, cognitive reappraisal, problem-solving, rumination, avoidance of emotions, and suppression. Functional cognitive strategies for emotion regulation are impaired in those with eating disorders, suggesting they are potential targets for intervention (47). For example, women with eating disorders, across ED subtypes, are inclined to suppress emotions and lack the capacity to reappraise emotions. Rumination, repetitive thoughts about negative experiences and emotions, is implicated in mood and anxiety disorders and there is a growing body of research on rumination in relation to eating disorders. In AN, rumination is particularly high around topics such as negative emotions and body-related cognitions (48).

Development of cognitive and behavioral strategies for managing emotions, once recognized, is central to the cognitive behavioral treatment (CBT) approach. In contrast, the way that FBT enhances these skills is perhaps less intuitive. As a starting point, though, FBT posits that the parents and family are the usual, natural, and potentially most effective way to help adolescents with AN learn about their emotions and how to manage them. In the first phase of FBT, the adolescent with AN displays a range of emotions—from a seeming unemotional stance to one that is highly dramatic and dysregulated, especially during mealtimes. Often the expression of these heightened emotions is new to both the patient and the family. The use of externalization helps the family to understand that these emotions are related to the disorder of AN, especially because they are expressed in contexts specifically involving demands for eating and weight gain. Externalization helps to reduce blame and judgment of the expression of these heightened emotions. The parents are helped to learn not to react to these outbursts and instead to tolerate them rather than to respond in kind or by giving in to food refusal. In this way, the adolescent is helped to learn to manage and regulate her emotions through the repeated modeling and support of parents, in the way tantrums in a much younger child respond to similar forms of containment.

Siblings can also play a role in helping to manage emotions in FBT by providing distractions and alternative supportive activities such as playing games or watching videos. Strategies for under emotional expression as well as depressed and withdrawn adolescents are similarly managed by parents with sibling support in phase 1. In phases 2 and 3, as the adolescent is eating more normally, emotional difficulties tend to be less about reactions to food and weight gain, and instead are about social and familial relationships. Anxieties about peer acceptance and sadness about the impact of AN on relationships is part of the focus of these phases. FBT sees the anxieties about self and others that “emerge” as weight and shape concerns recede as integral to the disorder rather than separate and new. The therapist identifies these issues and their specifics in the one to one meeting held before the whole family session and helps the adolescent to express these emotions with the family and together they develop plans to address them. Thus, management of these emotions is often treatment goals during the later phases of FBT.

### Cognitive Content and Processes

The cognitions of greatest relevance in maintaining the psychopathology of AN are those related to the value of weight and shape in self-evaluation (49). FBT does not include interventions to directly challenge these maladaptive thoughts and beliefs. In contrast, CBT for AN includes both motivational and cognitive restructuring techniques. These approaches directly address these problems and while conceptually of interest, data supporting their effectiveness for AN, and adolescent AN in particular are limited (50, 51). In FBT, the approach to addressing distorted beliefs and cognitions about food, weight and eating is initially approached behaviorally and indirectly. Data suggest that in FBT, changes in these distorted thoughts occurs about 6–8 months after weight restoration (6, 52). The timing is important here because this “lag” is likely related to prolonged reduction in the behaviors that maintain and reinforce these distorted cognitions. In other words, FBT contends that only with sustained weight normalization and continued inhibition of under eating, dieting, and over exercise do the cognitions lessen, presumably as a result of lack of reward for them over a prolonged period.

At the same time that FBT takes this longer view of cognitive change, there are a number of interventions in FBT that facilitate cognitive change and practice. For example, in phase 1, the adolescent is usually preoccupied with weight and shape concerns, but because in each session her weight progress is discussed openly and frankly, she must learn to manage her anxieties about weight gain because she is gradually, but persistently presented with anxiety provoking data about weight to increase acceptance and toleration of them (32, 53). By insisting on these exposures, even if they are sometimes emotionally challenging, FBT blocks avoidance. The therapist in the one on one time and the parents and family in session and at home can provide emotional support as the adolescent is learning to manage her cognitions about fear of weight gain. In sum, repeated exposure over the course of FBT leads to diminished weight gain fears. In phase 2, the adolescent is asked to participate actively with her parents in thinking through dietary and activity changes. In other words, she is engaged in problem solving around challenging her beliefs and their behavioral impacts. Although not in any way formal cognitive restructuring as used in CBT, problem solving in consultation with her parents lead to shifts in thinking in FBT.

A number of studies now support the importance of addressing thinking styles in the maintenance of AN through cognitive remediation therapy (CRT) (54). CRT targets metacognitive skills by helping patients recognize when their thinking style may be impacting decision-making and subsequent actions. In the case of AN, the most consistently demonstrated cognitive inefficiencies are found in set-shifting (changing track) and in central coherence (seeing the big picture in lieu of the detail) (55). These cognitive features are more marked in patients with AN who have high obsessive compulsive traits, traits which are known to moderate the outcome from FBT (36). This is an area where the role of starvation on cognitive function is also relevant. Fasting exacerbates set-shifting difficulties and impairs global processing, indicating weaker central coherence, in healthy controls (56). These findings (36) suggest that for some patients these cognitive inefficiencies may be secondary to malnutrition while in others malnutrition may enhance existing tendencies.

The evidence about cognitive processing problems in adolescents with AN is still developing (57). It appears that a good deal of cognitive rigidity improves with nutritional rehabilitation, so FBT's initial focus on this is a strategy to address cognitive flexibility. In preliminary studies CRT has been added to FBT in adolescents with AN and increased obsessionality (a marker for cognitive inflexibility) found some improvement but the effects were small (58). In general, in addition to weight gain, cognitive flexibility and big picture thinking are encouraged in FBT during phases 2 and 3 as the adolescent is asked to take, and is able to take, a broader perspective on her life and health than AN allowed and to participate in decision making about adolescent activities related not only to food, but to other social, educational, and family processes. Again, this differs significantly from the cognitive exercise strategies employed by CRT and likely helps with the majority of children with AN with very mild and likely state dependent cognitive processing difficulties (59). For those with more severe and persistent cognitive processing challenges, a course of CRT can be added without disrupting FBT (58). It is perhaps worth noting that the use of medications, particularly atypical antipsychotics, is suggested to address cognitions in AN. While such medications may be sometimes useful for the acute management of severe anxiety in AN, there is little data to support their effectiveness for improving cognitive content or process in AN itself (60).

### Social Communication and Connections

The emotional sensitivity of young people with AN appears to have particular relevance and specificity in the area of social interaction (61). Sensitivity to social rejection, such as falling out with friends, is at its peak in early adolescence, and is often cited as a trigger for onset of AN (62). Experimental studies suggest that adults with AN are hypervigilant to social rejection and avoidant of social reward. Added to this social sensitivity is increasing evidence that, at least for a proportion of those with AN, more pervasive social cognitive deficits may be a factor (63–65). Recent evidence that impaired theory of mind is also found in unaffected third-degree relatives of patients with AN supports the idea that these are trait rather than state characteristics (66, 67). Given the importance of social relationships in maintaining psychological health and wellbeing for young people, treatments, including FBT, should attend to reported difficulties in this area.

When presenting for FBT, adolescents with AN have often become socially isolated from families and friends. While there may have been social anxieties and difficulties prior to the onset of AN, these tend to be exacerbated by the illness. The preoccupations and activities required to maintain AN tend to reinforce social isolation. AN becomes the social world for many adolescents who are ill. The impact of social isolation on mental health, social and peer relationships, and overall adolescent development are potentially life altering, setting the adolescent on a life time course of a lonely life revolving around weight and eating (65). FBT takes the perspective therefore that curing AN is a necessary first step to addressing these socialization processes (68). Only by removing the behavioral and cognitive barrier that in AN leads to socially isolating the adolescent can progress be made. Parents and siblings are the first line of attack in helping the adolescent reengage with others. In phase 1, this means restoring healthy weight, meal time exposures with family, and over time at school. In phase 2, involvement with peers in an age appropriate manner, specifically around food and eating are stressed as a necessary step toward facilitating and developing peer relationships. For those who are in the early stages of adolescence, these may be novel experiments—eating on sleepovers, class birthdays, or parties. For older adolescents, this may mean eating on dates or in dining halls similar to those that would be found in boarding schools or universities. While the direct focus is on behavioral learning and there is a specific focus on food and eating, participation in socialized eating actually is more than this because it promotes overall social growth and normalization of peer relationships during phase 3. For a small number of patients, social difficulties is more marked, a factor thought to be important for prognosis and treatment response (69). It is important that these traits are identified and addressed. However, if developmental social learning processes are attended to alongside weight restoration, and reintegration with peers is achieved, the adolescent brain has potential to adapt and learn these skills in the majority of cases.

### Psychiatric Comorbidities

Comorbid psychiatric disorders are commonly diagnosed with AN, particularly depression, anxiety disorders, and obsessive compulsive disorder (53, 70–72). For many, these disorders though diagnosable are part of the clinical impact of AN on the adolescent. Starvation leads to depression, fears about weight gain and food increases anxiety, and obsessive thoughts about food, eating rituals, and exercise routines are often consistent with symptoms of OCD. In those cases, FBT's highly focused approach to the treatment of AN leads to marked decreases in these disorders without focusing on them directly (6, 73). For the most part, FBT recommends postponing other psychological treatments for comorbidity until AN is resolved and a healthy weight restored. This is recommended because of the life threatening nature of AN and the demands, both psychological and physical, on the adolescent and her family. This staged approach is feasible when the comorbidities are relatively mild or moderate. However, for patients with a clear history that predates AN, medications for comorbidities may well be helpful in the context of FBT.

Another comorbid problem that is sometimes a concern is posttraumatic stress disorder (PTSD). Trauma or other adverse events can trigger eating disorders if extreme enough, even in those who might otherwise have healthy emotional responses, such that distress becomes overwhelming (74). A severe life event or difficulty, generally of an interpersonal nature, was identified prior to onset in 67% of a clinical sample of cases of AN (75). Intrusive thoughts and related anxieties and fears related to these traumas can be an impediment to treatment, whatever the approach. FBT takes the position, however, FBT takes the position that traumas like other comorbidities should be treated independently after AN is mostly resolved because of the life-threatening and long term medical effects of ongoing starvation. Attempting to address trauma at the same time as weight restoration can lead to confusion about what the focus of treatment should be. A case example of this is included in the FBT to illustrate how FBT prepares the adolescent to address trauma without the complications (medical and psychological) of AN interfering and disrupting trauma work.

### Family Factors

While any suggestion that specific family “types” or processes are implicated in the causation of eating disorders remain unproven (76), the importance of family support and the responses of close others are key to recovery seems to be more certain (77). Problems in families include parental and sibling anxiety, worry, blame, criticism, or hostility, resulting in communications and behaviors which have been hypothesized to influence outcome (78), and may moderate treatment (79). The impact of these problems can be seen in common examples from clinical practice where the parental relationship, whether they are a couple or not, makes coparenting challenging. This can range from overt hostility in couples who are in the process of separating, to more subtle triangulation of young people in the parental relationship. Equally, the application of standard FBT can be impacted when a parent is grieving the loss of their partner from death or separation, or is unable by virtue of other demands (such as caring for others) or their own mental or physical health problems, to provide the necessary support to their child with AN. Data suggest the FBT may take longer in such cases (80). While there is evidence for the effectiveness of a parent only format for FBT in addressing families with these types of problems (81, 82), the usual whole family manual provides many examples of how to work with the family as a whole despite the presence of these issues. These include overtly identifying criticism, hostility, and nonalignment as impediments to effective behavioral management of AN, modeling a noncritical stance and illustrating noncritical comments through the extensive use of externalization and agnosticism to reduce blame and guilt.

## Concluding Comment

In this article we have identified a number of significant, commonly occurring problems in adolescent AN and described how the context, structure, and interventions of FBT can be used to successfully address many of them without introducing new content or augmentative treatments that to date have not been found systematically beneficial (83). Careful reading, accredited training, adequate supervision, and experience using manualized FBT would likely decrease concerns about how FBT helps with the full range of problems associated with adolescent AN. That said, there is no data available that support that FBT is superior to other psychotherapies in addressing these broader issues nor the impact of improvements in them on risk for relapse. Future studies are needed to compare the relative benefits of FBT to other approaches [e.g., CBT-E (50), Family Therapy-Anorexia Nervosa (84), Multi-Family Group Therapy (85), and Adolescent Focused Therapy (13)] on the broader psychopathology of AN. It may be particularly important to examine the relative effect of FBT compared to Systemic Family Therapy for adolescents with comorbid obsessive compulsive disorder as this was identified *post hoc* as a moderator favoring Systemic Family Therapy and should be confirmed (8). Furthermore, while FBT is effective for many, there is considerable room for improvement in outcomes and strategies to improve or augment FBT developed and tested (83). Careful study of the mechanisms mediating response to FBT, such as emotion regulation, trait characteristics, and social competence as well as parent factors as outlined above, may give clues to possible enhancements that would increase the proportion of patients responding to the intervention.

## Author Contributions

JL and DN conceptualized the paper and wrote the content.

## Conflict of Interest

JL receives royalties from Guilford Press and Routledge for books related to family-based treatment and is co-owner of the Training Institute for Child and Adolescent Eating Disorders that trains professionals in family-based treatment.

The remaining author declares that the research was conducted in the absence of any commercial or financial relationships that could be construed as a potential conflict of interest.
